# Granzyme B deficiency promotes osteoblastic differentiation and calcification of vascular smooth muscle cells in hypoxic pulmonary hypertension

**DOI:** 10.1038/s41419-018-0315-5

**Published:** 2018-02-14

**Authors:** Min Mao, Min Zhang, Anqi Ge, Xin Ge, Rui Gu, Chen Zhang, Yao Fu, Jiayin Gao, Xiaoying Wang, Yang Liu, Daling Zhu

**Affiliations:** 1 Department of Biopharmaceutical Sciences, College of Pharmacy, Harbin Medical University (Daqing), Daqing, 163319 China; 2 Biopharmaceutical Key Laboratory of Heilongjiang Province, Harbin, 150081 China; 3 Department of Epidemiology, School of Public Health, Harbin Medical University, Harbin, 150081 China; 4 Department of Obstetrics and Gynecology, The Second Affiliated Hospital of Harbin Medical University, Harbin, China; 5 Department of Pharmaceutical Analysis, College of Pharmacy, Harbin Medical University, Harbin, 150081 Heilongjiang Province China

## Abstract

Calcification is a major risk factor for vascular integrity. This pathological symptom and the underlying mechanisms in hypoxic pulmonary artery hypertension remain elusive. Here we report that pulmonary vascular medial calcification is elevated in pulmonary artery hypertension models as a result of an osteoblastic phenotype change of pulmonary arterial smooth muscle cells induced by hypoxia. Notably, inhibiting store-operated calcium channels significantly decreased osteoblastic differentiation and calcification of pulmonary arterial smooth muscle cells under hypoxia. We identified granzyme B, a major constituent of cytotoxic T lymphocytes/natural killer cell granules involved in apoptosis, as the main regulator of pulmonary arterial calcification. Overexpression of granzyme B blocked the mineralization through its effect on store-operated calcium channels in cultured pulmonary arterial smooth muscle cells under hypoxic conditions. Mice with overexpression of granzyme B exposed to hypoxia for 3 weeks showed attenuated vascular calcification and pathological progression of hypoxic pulmonary arterial hypertension. Our findings emphasize the central function of granzyme B in coordinating vascular calcification in hypoxic pulmonary arterial hypertension.

## Introduction

Hypoxic pulmonary arterial hypertension (HPAH) is characterized by functional and structural changes in the pulmonary vasculature, leading to increased pulmonary vascular resistance and remodeling and right ventricular hypertrophy^1,2^. Multiple cellular dysfunctions are involved in pathological changes in the pulmonary artery, such as endothelial dysfunction, activation of fibroblasts, and smooth muscle cells (SMCs), crosstalk between cells within the vascular wall, and recruitment of circulating progenitor cells^3,4^. Vascular calcification is a major contributor to morbidity and mortality in patients with atherosclerosis, chronic kidney disease, and diabetes mellitus^5–7^. Medial vessel calcification results in increased vessel wall stiffness and decreased vessel compliance, which leads to increased arterial pulse wave velocity and pulse pressure, and eventually affects coronary artery perfusion and heart function^7,8^. Arterial calcification accounted for 13% of patients with pulmonary hypertension according to a previous clinical report^9^. These findings have led to the important questions of what mechanisms regulate pulmonary vascular calcification and what cell type give rise to the skeletal elements found in pulmonary vascular calcification.

In the vessel wall, SMCs are the predominant cell type and are essential for the structural and functional integrity of the vessel, which differentiate from a contractile to osteoblastic phenotype in response to pathological signals, such as inflammatory cytokines or mineral imbalance^8,10^. The induction of an osteogenic phenotype is characterized by an increase in the levels of osteogenic markers in the vascular wall, including runt-related transcription factor (Runx)2, bone morphogenetic protein (BMP)2, msh homeobox (MSX)2, or SRY-box (SOX)9, and the loss of SM22α. Calcifying SMCs generate matrix vesicles, or small extracellular membranous bodies, serve as mineral nucleation sites and are responsible for the initial deposition of calcium and phosphate in blood vessels^7,11^. Despite the importance of phenotypic change of SMCs, little is known of the mechanisms. Definitive studies on the regulation of osteoblastic differentiation and calcification of SMCs in HPAH are needed.

Granzyme B (GZMB) is a major constituent of cytotoxic T lymphocytes/natural killer (NK) cell granules, promoting apoptosis through proteolysis of a small number of substrates^12,13^. It also has an important role in several chronic inflammatory diseases such as atherosclerosis and chronic obstructive pulmonary disease^14,15^. Extracellular GZMB cleavage of decorin, biglycan, and betaglycan leads to the release of transforming growth factor-β1 from the matrix, suggesting that GZMB indirectly affects normal cell function by altering growth factor bioavailability^16^. Previous study unveiled a close relationship between the upregulation of GZMB and human calcific aortic valve disease, indicating an important role of GZMB in vascular calcification^17^. We suspect that GZMB regulates a shift of phenotype in vascular SMCs (VSMCs), leading to pulmonary artery calcification.

In the current study, we examined the medial vascular calcification and the underlying regulation mechanisms in hypoxic pulmonary hypertension. We showed that deposits of calcium mineral and expression of vascular calcification makers were elevated in response to hypoxia. Store-operated calcium channels (SOCCs) serve as the main source of calcium mineral. Moreover, we identified a novel role of GZMB deficiency, which has a critical role in SMC mineralization through an effect on SOCCs.

## Results

### Increased vascular medial calcification in PAH

**Fig. 1:**
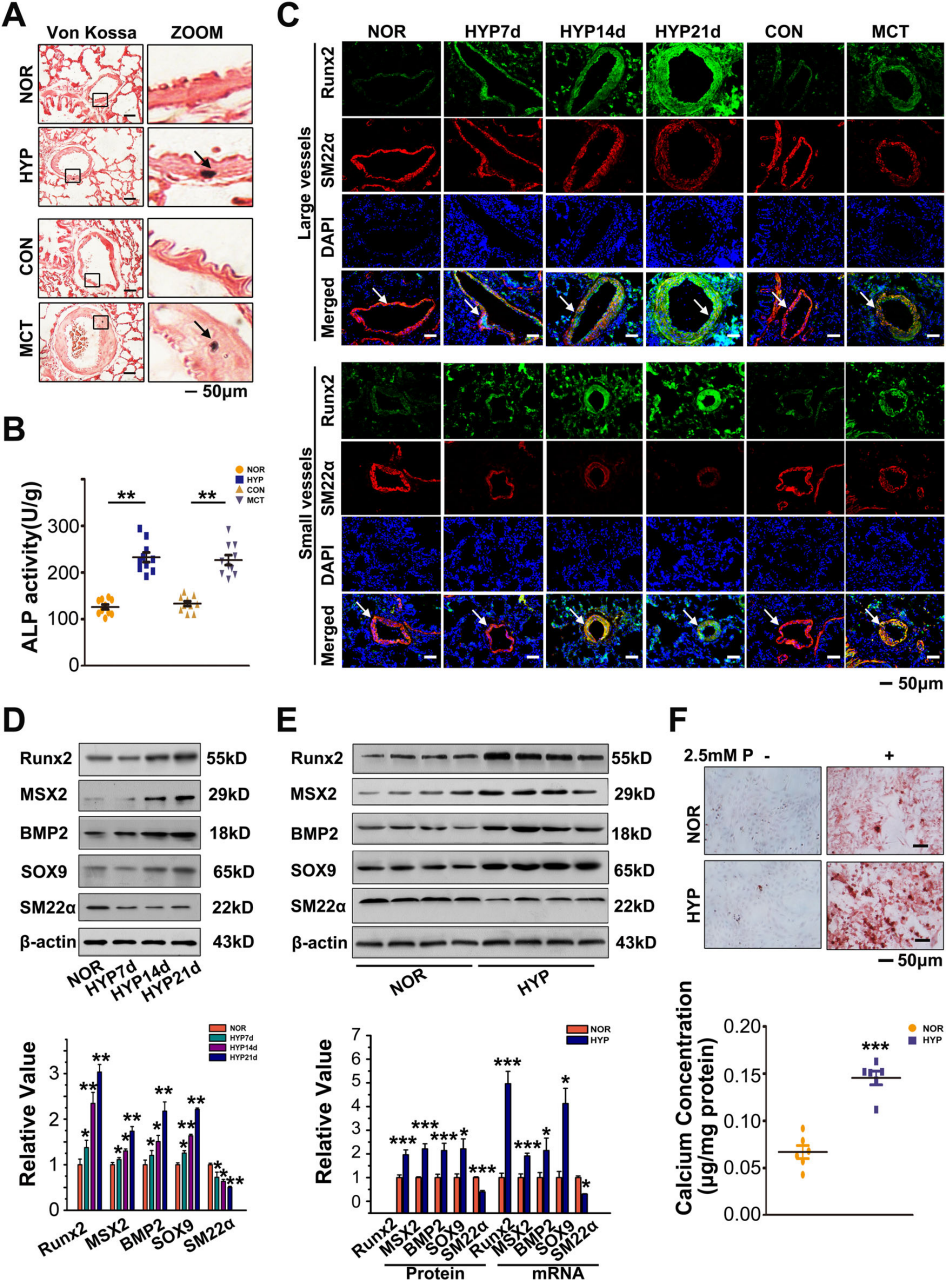
Increased vascular calcification in PAH. **a** Von Kossa staining revealed obvious deposits of calcium mineral in the medial vessels from the PAH rat model induced by chronic hypoxia or MCT. All panels are at × 20 magnification except for the zoomed ones. Scale bars = 50 μm; *n* = 10. **b** Chronic hypoxia or MCT administration increased activity of ALP in pulmonary arteries, *n* = 10. **c** Immunohistochemical analysis of cellular expression of Runx2 at the medial layer in lung tissues from normoxia, chronic hypoxia, and MCT-treated rats. All panels are at 20 × magnification. Scale bars = 50 μm; *n* = 10. **d** Western blotting of Runx2, MSX2, BMP2, SOX9, and SM22α expression in lung tissues from normoxia, chronic hypoxia, and MCT-treated rats. β-Actin served as the standard; *n* = 10. **e** PASMCs were exposed to hypoxia for 24 h and expression of Runx2, MSX2, BMP2, SOX9, and SM22α was evaluated with western blotting and real-time PCR. β-Actin served as the standard for western blotting; 18 s served as the standard for real-time PCR; *n* = 6. **f** PASMCs were cultured under hypoxia for 7 days upon treatment with procalcifying media containing 2.5 mM inorganic phosphate. Deposits of calcium mineral were assessed by Alizarin Red S staining **f** and determination of calcium content **g**; *n* = 6. Data are represented as mean ± SEM. *P < 0.05 and **P < 0.01 versus normoxia group. HYP, hypoxia, MCT, monocrotaline; NOR, normoxia

The rat models of pulmonary hypertension induced by chronic hypoxia and monocrotalin (MCT) were constructed to characterize the behavior and main localization of pulmonary vascular calcification. Von Kossa staining revealed obvious deposits of calcium mineral in medial vessels from PAH rat models (Fig. 1a). Moreover, activity of alkaline phosphatase (ALP) was increased in pulmonary arteries from PAH rat models (Fig. 1b). Runx2 is the key osteogenic regulator of osteoblastic differentiation and chondrocyte maturation^18^, and was increased in pulmonary arteries from PAH rat models (Fig. 1c, d). Concomitant with the onset of calcification, SMCs underwent anosteochondrogenic phenotype change also characterized by the loss of SMC markers (SM22α) (Fig. 1c). Besides, molecules regulating osteoblastic and chondrocytic differentiation, such as BMP2, MSX2, and SOX9, were also activated in vivo and in vitro (Fig. 1d, e). Obvious deposits of calcium mineral were also increased in vitro (Fig. 1f). Pulmonary arterial SMC (PASMC) mineralization was facilitated by hypoxia and led to increased deposits of calcium mineral.

### Inhibition of SOCC attenuated PASMC mineralization induced by hypoxia

**Fig. 2:**
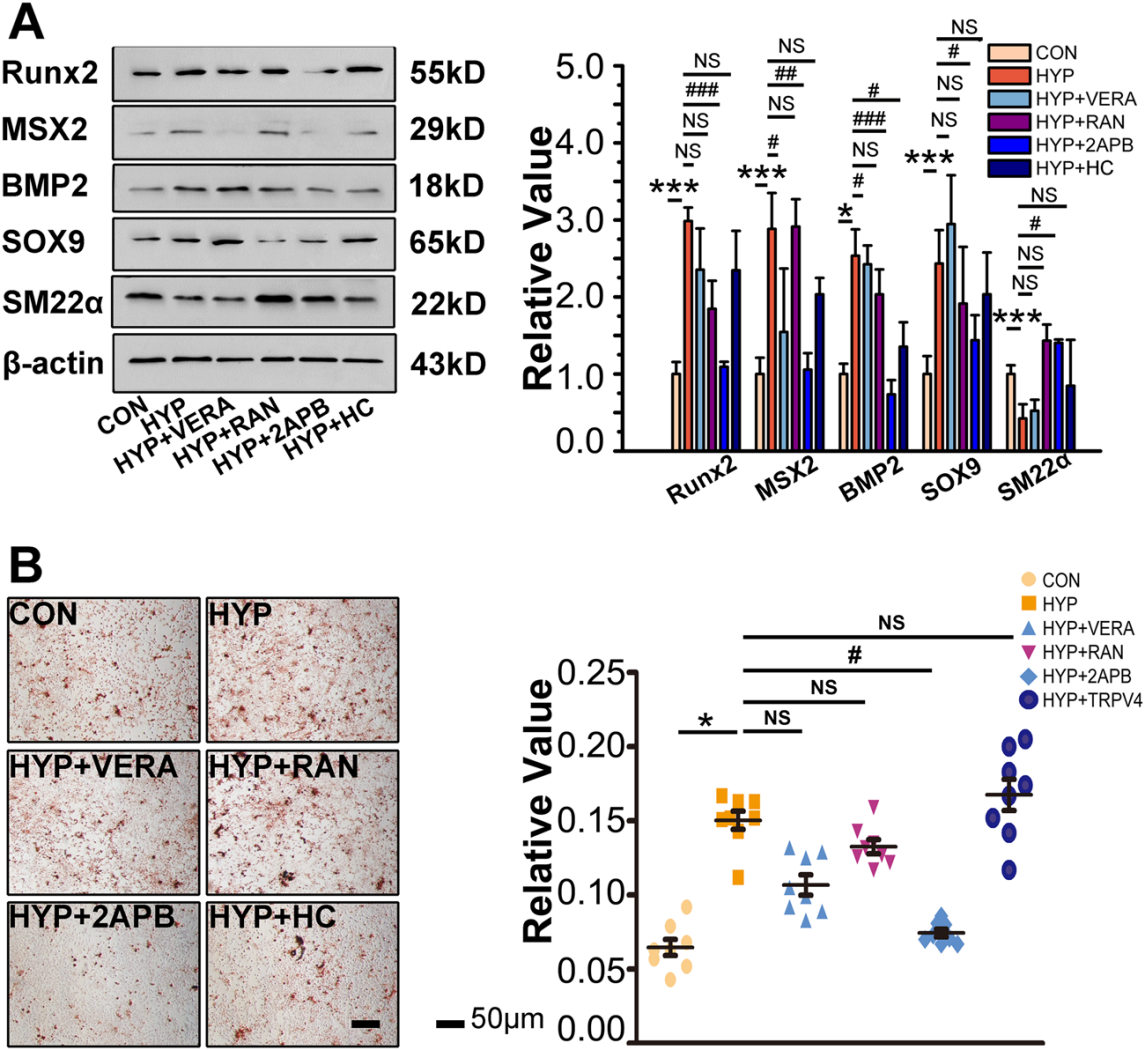
Inhibition of SOCCs attenuated PASMC calcification induced by hypoxia. **a** PASMCs pretreated with verapamil HCl (5 μM), ranolazine 2 HCl (100 μM), 2-APB(10 μM), or HC067047 (10 μM), PASMCs were exposed to hypoxia for 24 h. Expression of Runx2, MSX2, BMP2, SOX9, and SM22α was evaluated with western blotting. β-Actin served as the standard; *n* = 8. **b** PASMCs pretreated with verapamil HCl, ranolazine 2 HCl, 2-APB, or HC067047 were cultured under hypoxia for 7 days upon treatment with procalcifying media containing 2.5 mM inorganic phosphate. Vascular calcification was assessed by Alizarin Red S staining **b** and determination of calcium content **c**; *n* = 8. Data are represented as mean ± SEM. *P < 0.05 and **P  < 0.01 versus normoxia group. ^#^*P* < 0.05, ^##^*P* < 0.01, and ^###^*P* < 0.001 versus hypoxia group. HC, HC067047; HYP, hypoxia; NOR, normoxia; RAN, ranolazine 2HCl; VERA, verapamil HCl

To explore the specific source of the deposits of calcium mineral under hypoxia, verapamil (inhibitor of l-type calcium channels), ranolazine (inhibitor of sodium/calcium channels), 2-APB (inhibitor of SOCCs), and HC067047 (inhibitor of transient receptor potential cation channel subfamily V member 4 (TRPV4)) were administered in vitro. The altered expression of Runx2, MSX2, BMP2, SOX9, and SM22α under hypoxia was not significantly reversed by verapamil, ranolazine, or HC067047, nor were the deposits of calcium mineral. 2-APB was the most effective agent for attenuation of anosteochondrogenic phenotype change of PASMCs and the increased deposits of calcium mineral (Fig. 2a, b). These data suggest that SOCCs are the main channel for PASMC mineralization under hypoxia.

### Deficient expression of GZMB in PAH

**Fig. 3:**
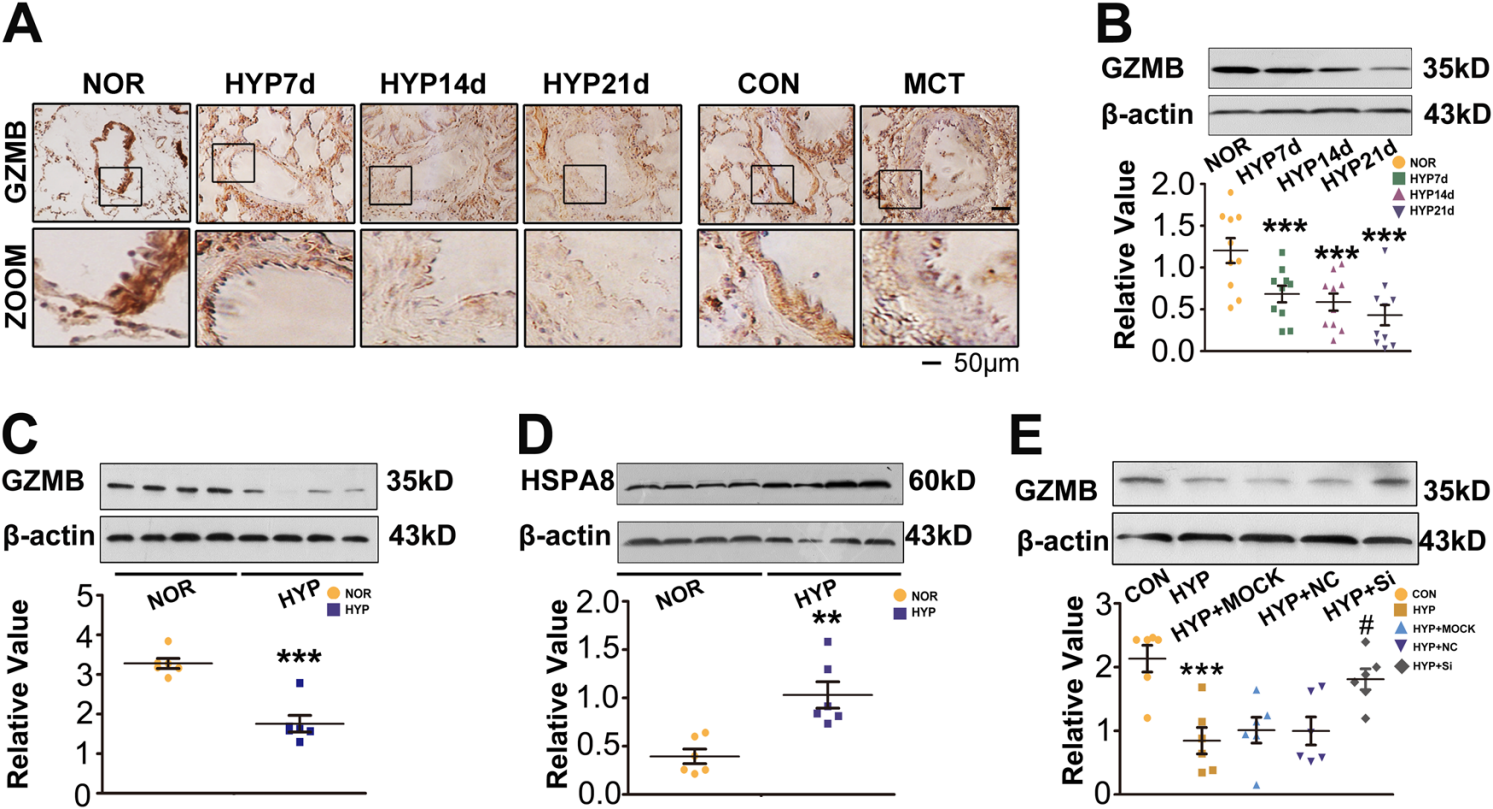
Expression of GZMB was abnormally decreased in PAH. **a** The cellular location of GZMB was analyzed immunohistochemically, revealing the medial layer as the location of GZMB. All panels are at × 20 magnification except for the zoomed ones. Scale bars = 50 μm; *n* = 10. **b** Expression of GZMB was evaluated with western blotting in lung tissues from the normoxia, chronic hypoxia, and MCT-treated rats. β-Actin served as the standard; *n* = 10. **c** PASMCs were exposed to hypoxia for 24 h and expression of GZMB was evaluated with western blotting. β-Actin served as the standard; *n* = 6. **d** PASMCs were exposed under hypoxia for 24 h and expression of HSPA8 was evaluated with western blotting. β-Actin served as the standard; *n* = 6. **e** PASMCs were pretreated with si-HSPA8, expression of GZMB was evaluated with western blotting. β-Actin served as the standard; *n* = 6. Data are represented as mean ± SEM. *P < 0.05, **P < 0.01, and ***P < 0.001 versus normoxia group. ^#^*P* < 0.05, ^##^*P* < 0.01, and ^###^*P* < 0.001 versus hypoxia + negative control group. CON, control; GZMB, granzyme B; HYP, hypoxia; IP, immunoprecipitation; MCT, monocrotaline; MOCK, transfection reagent; NC, negative control; NOR, normoxia.

The above findings led us to investigate the mechanisms for regulating the SOCCs and pulmonary vascular calcification. The cellular location of GZMB was revealed by immunohistochemistry and medial vessels were the main location of GZMB (Fig. 3a). Deficient expression of GZMB was confirmed at the posttranslational level in lung samples from chronic hypoxic rats in a time-dependent manner (Fig. 3b). Compared with the high expression of GZMB under normal conditions, the expression of GZMB was decreased under hypoxic conditions in PASMCs (Fig. 3c).

We attempted to gain insights into the mechanisms of deficient expression of GZMB. Earlier reports suggest that GZMB is degraded by autophagy^19^. Considering that chaperon-mediated autophagy (CMA) was more selective in degrading target proteins, we evaluated the cellular location and expression of heat shock cognate 71 kDa protein (HSPA8) and lysosome-associated membrane glycoprotein (LAMP) 2 A, key regulators of CMA. Importantly, both HSPA8 and LAMP2A were located in medial vessels and activated in vivo and in vitro under hypoxia (Fig. 3d and S2A–E). Immunofluorescence staining and co-immunoprecipitation assay were conducted to verify the interaction between HSPA8 and GZMB (Fig. S2F, G). To elucidate the role of CMA induced by HSPA8, expression of HSPA8 was knocked down with its specific small interfering RNA (siRNA). The altered expression of GZMB under hypoxia was blocked by HSPA8 siRNA (Fig. 3e and S2H). Expression of GZMB was decreased in the medial vessels in PAH, which resulted from activation of CMA process under hypoxia.

### Enforced expression of GZMB counteracted the osteoblastic differentiation and calcification of PASMCs under hypoxia

**Fig. 4:**
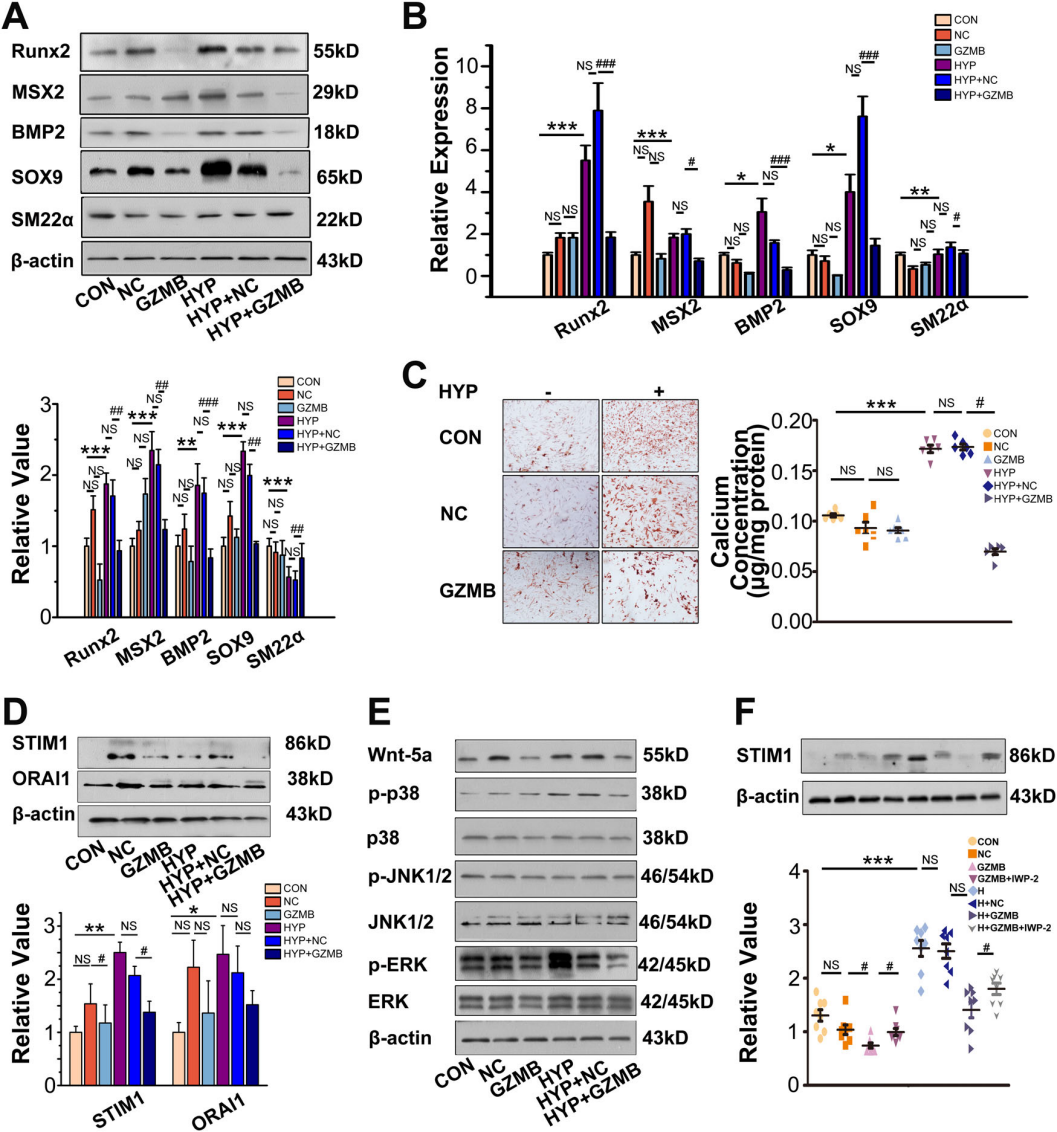
GZMB counteracted osteoblastic differentiation and calcification of VSMCs under hypoxia. **a** Plasmid aimed at overexpression of GZMB was transfected into PASMCs and then exposed to hypoxia for 24 h. Expression of Runx2, MSX2, BMP2, SOX9, and SM22α was evaluated with western blotting. β-Actin served as the standard; *n* = 8. **b** Expression of Runx2, MSX2, BMP2, SOX9, and SM22α was evaluated with real-time PCR; 18 s served as the standard; *n* = 6. **c** PASMCs were cultured under hypoxia for 7 days upon treatment with procalcifying media. Vascular calcification was assessed by Alizarin Red S staining and determination of calcium content; *n* = 6. **d** GZMB plasmid was transfected into PASMCs and exposed to hypoxia for 24 h. Expression of STIM1 and ORAI1 was evaluated with western blotting. β-Actin served as the standard; *n* = 8. **e** Overexpression of GZMB was achieved with plasmid and several classical signaling pathways were evaluated with western blotting. β-Actin served as the standard; *n* = 6. **f** IWP-2 (27 nM) was administered to inhibit the non-canonical Wnt signals and expression of STIM1 was evaluated with western blotting. β-Actin served as the standard; *n* = 8. Data are represented as mean ± SEM. *P < 0.05, **P < 0.01 and ***P < 0.001 versus normoxia group. ^#^*P* < 0.05, ^##^*P* < 0.01 and ^###^*P *< 0.001 versus hypoxia + negative control group. CON, control; ZMB, granzyme B plasmid; HYP, hypoxia; GNC, negative control; NOR, normoxia

To determine whether PASMC osteogenic differentiation coincided with the deficiency of GZMB, we administered a plasmid that aimed at enforcing expression of GZMB. Overexpression of GZMB blocked the anosteochondrogenic phenotype change of PASMCs characterized by the increase in SM22α and loss of osteochondrogenic markers (Runx2, MSX2, BMP2, and SOX9) (Fig. 4a, b). The increased deposits of calcium mineral by hypoxia were attenuated by GZMB plasmid (Fig. 4c).

Considering the SOCCs as the main source of PASMC mineralization, we investigated whether expression of stromal interaction molecule (STIM) 1 and calcium release-activated calcium modulator (ORAI) 1, the key regulators of SOCCs, were affected by GZMB. Enforced expression of GZMB inhibited activation of STIM1, but no significant differences were observed in the expression of ORAI1 (Fig. 4d). To ascertain the signaling pathways for GZMB inhibition of STIM1, expression of Wnt5a, mitogen-activated protein kinase and phosphoinositide 3-kinase was evaluated by western blotting after GZMB plasmid administration. Enforced expression of GZMB significantly inhibited expression of Wnt5a (Fig. 4e and S3). Administration of IWP-2, an inhibitor of non-canonical Wnt signals, reversed the effect of GZMB on STIM1 (Fig. 4f). Overexpression of GZMB blocked the osteogenic differentiation of PASMCs under hypoxia and this effect was mediated by activating non-canonical Wnt signals, which restored expression of STIM1 and SOCCs.

### SM22α promoter-driven overexpression of GZMB mice display inhibited vascular medial calcification and repressed indices of PAH in the chronic hypoxia model

**Fig. 5:**
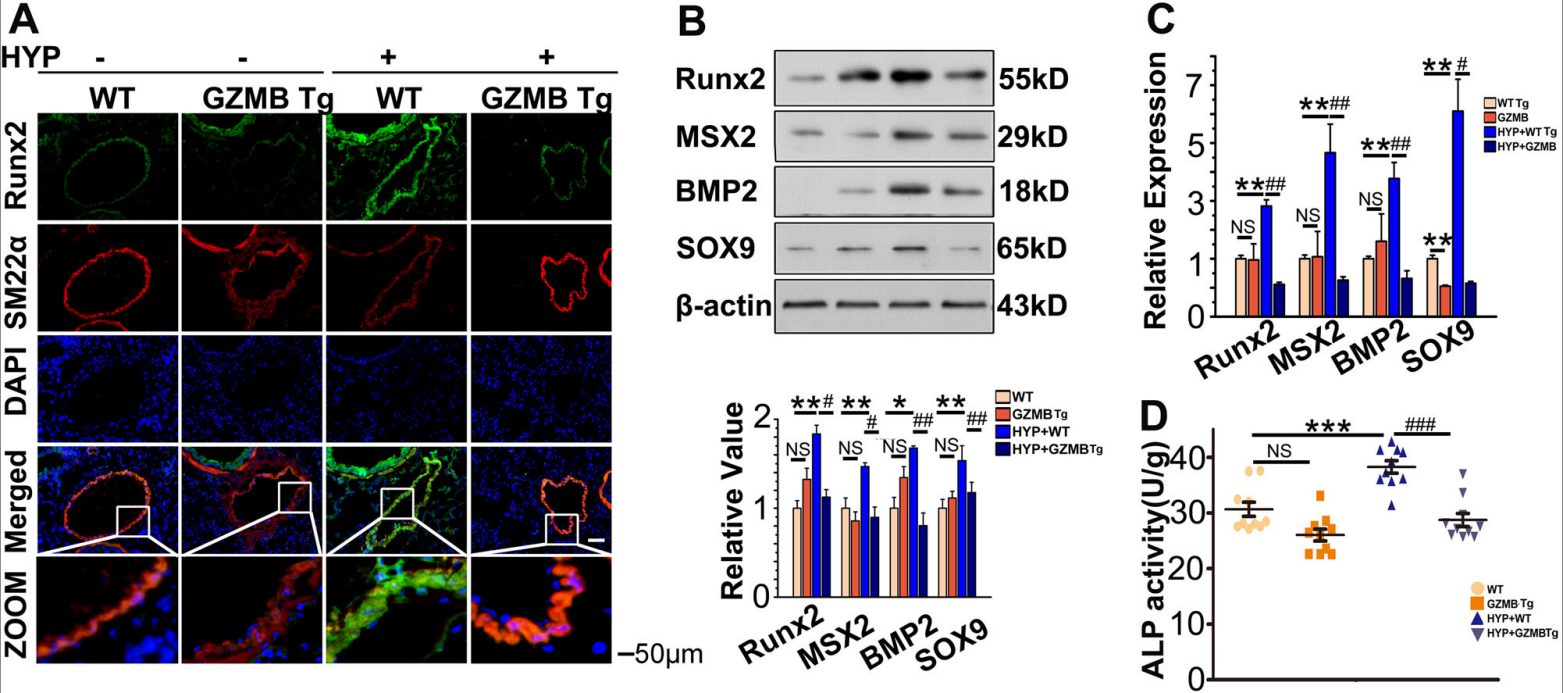
Transdifferentiation of PASMCs was blocked in SM22α-GZMB Tg mice. **a** Immunohistochemical analysis of Runx2 in lung tissues from wild-type and SM22α-GZMB Tg mice after hypoxia and normoxia for 3 weeks. All panels are at × 20 magnification. Scale bars = 50 μm; *n* = 10. **b** Expression of Runx2, MSX2, BMP2, and SOX9 was evaluated with western blotting in lung tissues from wild-type and SM22α-GZMB Tg mice after hypoxia and normoxia for 3 weeks. β-Actin served as the standard; *n* = 10. **c** Real-time PCR was administered to estimate the mRNA level of Runx2, MSX2, BMP2, and SOX9; 18 s served as the standard; *n* = 6. **d** Activity of ALP was evaluated with pulmonary arteries from wild-type and SM22α-GZMB Tg mice after hypoxia and normoxia for 3 weeks. *n* = 10; data are represented as mean ± SEM. *P < 0.05, **P < 0.01, and ***P < 0.001 versus wild-type group. ^#^*P* < 0.05, ^##^*P *< 0.01, and ^###^*P* < 0.001 versus hypoxia + wild type group. HYP, hypoxia; WT, wild type

We next investigated the role of GZMB in vascular medial calcification during development of PAH. SM22α-GZMB transgenic (Tg) mice exposed to normoxia did not differ in expression of osteochondrogenic markers compared with their wild-type littermate controls (Fig. 5a–c). In contrast, SM22α-GZMB Tg mice exposed to hypoxia for 3 weeks revealed significantly reduced osteochondrogenic markers compared with their littermate controls (Fig. 5a–c). We also found that activity of ALP was significantly depressed in SM22α-GZMB Tg mice exposed to hypoxia for 3 weeks (Fig. 5d).

**Fig. 6:**
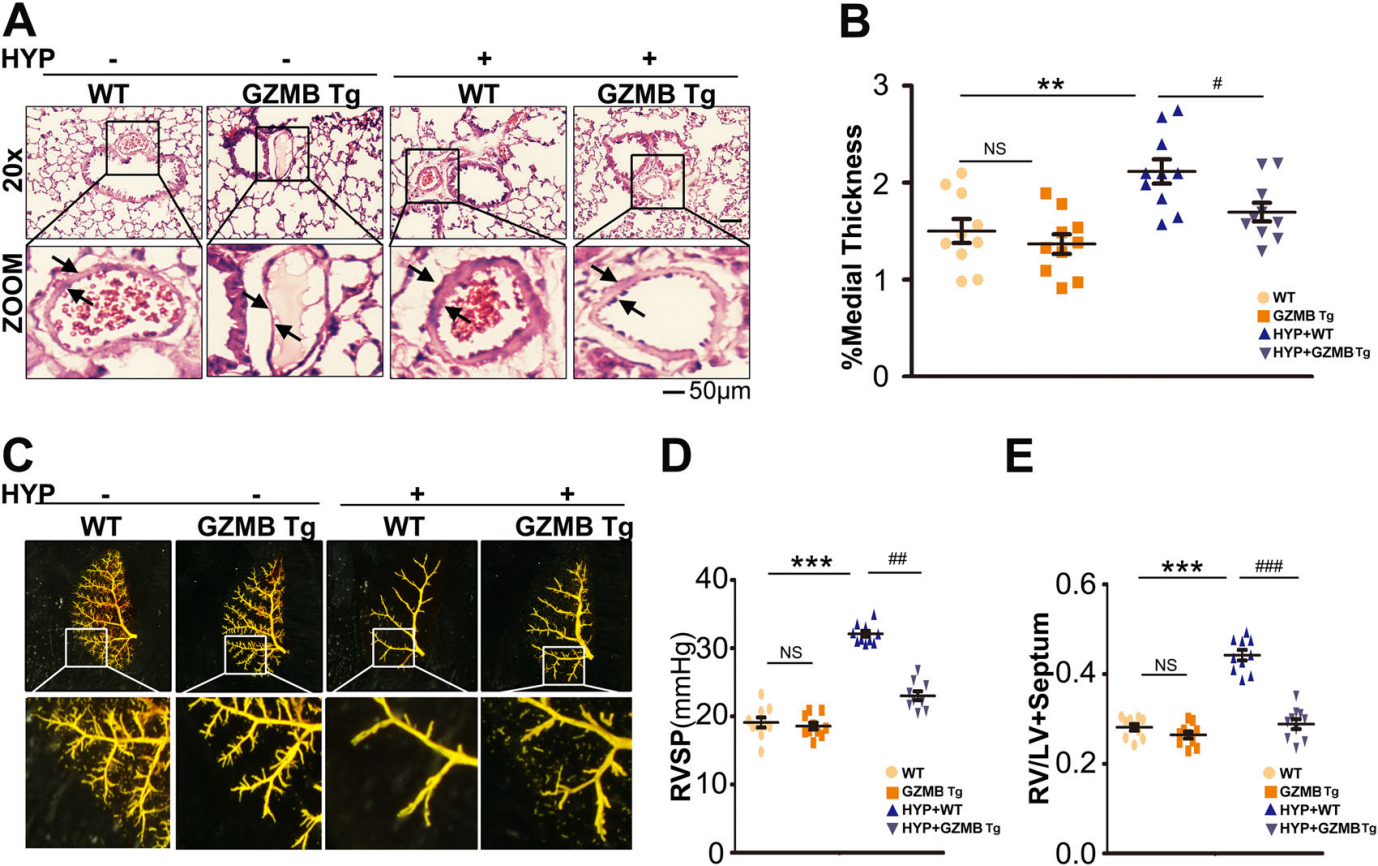
Progression of PAH was partially inhibited in SM22α-GZMB Tg mice. **a** SM22α-GZMB Tg mice showed reversal of the wall thickening **a**, **b**, depressed pulmonary vascular density **c**, mean RVSP **d**, and indices of RV weight (RV/LV+Sep) **e**, induced by chronic hypoxia. All panels are at × 20 magnification. Scale bars = 50 μm; *n* = 10. Data are represented as mean ± SEM. *P < 0.05, **P < 0.01, and ***P < 0.001 versus wild type group. ^#^*P* < 0.05, ^##^*P *< 0.01, and ^###^*P *< 0.001 versus hypoxia + wild-type group. HYP, hypoxia; WT, wild type

To evaluate further the functional role of GZMB during development of PAH, we used SM22α-GZMB Tg mice and littermate controls. We performed a morphological assay to analyze vascular remodeling in SM22α-GZMB Tg mice. Under normoxic conditions, SM22α-GZMB Tg mice displayed similar medial thickness relative to their littermate controls. Exposure to 3 weeks’ hypoxia increased medial thickness ~ 1.6-fold relative to baseline and this value was significantly higher than that of hypoxia-exposed SM22α-GZMB Tg mice (Fig. 6a, b). In addition, SM22α-GZMB Tg mice exposed to hypoxia displayed restored density of pulmonary vasculature and right ventricular systolic pressure compared with their littermate controls (Fig. 6c–e).

Given that PAH induces RV hypertrophy and heart failure, we evaluated cardiac function by echocardiography. SM22α-GZMB Tg mice under hypoxia exhibited remarkable RV hypertrophy and a decreased ratio of PA acceleration time/ejection time, indicating PA diastolic dysfunction (Fig. S4A, B). However, left ventricular (LV) function of SM22α-GZMB Tg mice was similar to that of wild-type mice, suggesting that PAH in SM22α-GZMB Tg mice is not secondary to LV dysfunction (Fig. S4C). Vascular contractility was determined by evaluation of expression of myosin. Pulmonary arteries from SM22α-GZMB Tg mice exposed to hypoxia exhibited restored expression of myosin (Fig. S4D, E).

## Discussion

We believe this is the first study to examine the underlying mechanisms of vascular calcification in HPAH. There are three principal findings of this study that help advance our understanding of pulmonary arterial medial calcification in HPAH: (1) SOCCs are the main source of the deposits of calcium mineral under hypoxia; (2) GZMB signaling has a vital role in regulating the osteoblastic response within the SOCCs; and (3) activation of CMA serves as the main process for degradation of GZMB (Fig. S8).

In a clinical computed tomography study, 13% of PAH patients were reported of pulmonary arterial calcification^9^. Such symptoms were also reported in Ruffenach G’s recent study; however, the regulatory mechanisms remain unknown^20^. We identified this pathological observation and its main location in HPAH. In the established PAH rat models, significant deposits of calcium mineral were detected in the pulmonary arterial vasculature (Fig. 1a). Master osteoblast transcription factor Runx2 along with concomitant downregulation of SMC contractile proteins were located in the pulmonary artery media (Fig. 1c). In addition, hypoxia treatment promoted expression of bone-related proteins (Runx2, MSX2, BMP2, and SOX9) in vitro, demonstrating osteoblastic differentiation and calcification of PASMCs. Medial vessel calcification results in increased vessel wall stiffness and decreased vessel compliance, which leads to increased arterial pulse wave velocity and pulse pressure, and eventually affects coronary artery perfusion and heart function^7,8^. Identifying the main localization and the underlying mechanisms of the basic calcification process in HPAH is of great importance in searching for the therapeutic target for HPAH.

Intracellular Ca^2+^ is involved in multiple pathophysiological processes and tightly regulated in VSMCs by calcium channels, including l-type voltage-dependent calcium channels, sodium/calcium channels, SOCCs, and TRPV4^21^. Accumulating evidences demonstrated calcium sources in VSMCs as critical components during vascular calcification^22,23^. Chen and colleagues^24^ have reported the potential role of verapamil in regulating the calcification of bovine VSMCs. We found that inhibiting SOCCs significantly decreased PASMC mineralization (Fig. 2), which suggests that increased PASMC osteogenic differentiation in response to hypoxia may be induced via activation of SOCCs. Our results differ from the previous studies. We speculate that the difference between systemic and pulmonary circulation cause lower efficiency of verapamil under hypoxia. Importantly, the possible roles of diverse calcium channels are not explored in earlier reports and the results from our study suggest a new means for exploring the main source for vascular calcification.

GZMB is the main cytotoxic agent released by T lymphocytes upon activation or encounter with foreign antigens in the periphery^25^. Our results indicate that GZMB is significantly decreased under hypoxia, which promotes phenotypic change and leads to osteoblastic differentiation and calcification of PASMCs. SM22α-GZMB Tg mice displayed inhibited vascular calcification and depressed progression of HPAH (Figs. 5 and 6). It is generally accepted that apoptosis provides the matrix for calcification and there is a close relationship between apoptosis and calcification. In our study, instead of effecting phenotypic change and mineralization of PASMCs through activating apoptosis (Fig. S5), GZMB achieved this end mainly through affecting STIM1, the key sensor of endoplasmic reticulum (ER) Ca^2+^. Depletion of ER Ca^2+^ contributes to Ca^2+^ entry, a process termed store-operated Ca^2+^ entry (SOCE)^24,26^. STIM1-dependent SOCE is observed in a wide variety of cell types including platelets, skeletal muscle cells, and keratinocytes^27,28^. Our results further demonstrate that the effect of GZMB on STIM1 is mainly achieved by inhibiting the Wnt signaling pathway (Fig. 4e, f). All these results reveal the important role and regulatory mechanism of GZMB in pulmonary vascular calcification, which provide a novel clue for detecting treatment of HPAH. Moreover, bromodomain protein (BRD)-4 and hypoxia inducible factor (HIF)-1 are recognized to have an important role in calcification and HPAH. Thus, we investigate whether GZMB deficiency influences the expression of BRD4 and HIF-1^29,30^. We observed a decrease in HIF-1 protein levels when the expression of GZMB was enforced (Fig. S7). The role of HIF-1 in GZMB eficiency-induced vascular calcification still needs more exploration. Interestingly, no obvious changes were detected in BRD4 protein levels (Fig. S7). BRD4 may have an effect in pulmonary vascular calcification but in different axis.

While exploring the mechanisms of deficient expression of GZMB, we found that CMA was also activated in response to hypoxic treatment and specifically recognizes GZMB, which results in degradation and dysfunction of GZMB. CMA is a proteolytic mechanism that contributes to degradation of intracellular proteins in lysosomes. Proteins that undergo degradation by CMA are selected individually through a recognition motif in their amino acid sequences. This selectivity of CMA permits timed degradation of specific proteins with regulatory purposes supporting a modulatory role for CMA in enzymatic metabolic processes and subsets of the cellular transcriptional program. Activated CMA is also linked to HIF-1A degradation in cancer cells, which compromises the ability of cancer cells to respond to and survive hypoxia, suggesting the complex pathophysiological effect of CMA under hypoxic conditions^31,32^.

There are limitations. Accumulating evidence demonstrated a close relationship between DNA damage signaling and SMC osteogenic differentiation^33^. DNA damage response may be a therapeutic target for blocking progression of vascular calcification^34^. Given GZMB has a role in inducing DNA damage, DNA damage may also have an effect in GZMB deficiency-induced pulmonary vascular calcification. Besides, Ozaki et al.^35^ detected Runx2 was involved in p53-dependent DNA damage response in collaboration with HDAC6. All these findings suggest there may exist a regulation loop among GZMB, Runx2, HDAC6, DNA damage, and vascular calcification. Detailed characterization of the relationships will be the focus of future studies.

Our findings shed light on a new function of GZMB in the regulation of pulmonary arterial calcification of HPAH. Our data suggest the working model shown in Fig. S8, in which CMA process degrades GZMB, which affects SOCCs to promote pulmonary vascular calcification in vitro and in vivo. This provides insights into a novel pathway that may be crucial in mediating pulmonary medial calcification in HPAH patients.

## Materials and methods

### Animals and lung tissue preparation

In accordance with the guidelines for the Care and Use of Laboratory Animals approved by the Institutional Animal Care and Use Committee, all experimental procedures in animals were carried out and conducted in compliance with the NIH guidelines. All surgery was carried out under sodium pentobarbital anesthesia and pain was minimized.

Adult male Sprague–Dawley rats with a mean weight of 130 g were obtained from the Harbin Medical University Experimental Animal Center. All animals were housed in accordance with the guidelines on preclinical research in PAH as described in the study by Bonnet et al.^36^. To explore the pathological process of HPAH, rats were randomly assigned to normoxia and hypoxia groups with fractional inspired oxygen (*F*i,_O2_) 0.21 and 0.12, respectively, according to previous studies^37–41^. The hypoxia group was maintained under hypoxic conditions (*F*i,_O2_ 0.12) for 7, 14, or 21 days in a normobaric environmental chamber. In the PAH model induced by MCT, rats received a single subcutaneous injection of 40 mg/kg MCT (37024, Sigma, St. Louis, MO, USA)^41^. At the end of the exposure period, the rats were anesthetized with pentobarbital injection (120 mg/kg, i.p.), then the thoraxes were opened and the lungs were quickly removed and processed for immunocytochemistry and immunofluorescence, as previously described^38,41^.

SM22α promoter-driven overexpression of GZMB C57BL/6 mice was purchased from Beijing Vitalstar Biotechnology (Beijing, China). The mice were bred and maintained at Harbin Medical University. The mice were 6 weeks old when the identification of genotypes was conducted. Eight- to 12-week-old mice with overexpression of GZMB were selected for functional experiments. The mice were fed a γ-irradiated sterile diet and autoclaved distilled water.

### Cell culture

The primary pulmonary arteries were derived from the adult Wistar rats (100–150 g) under microscope. The scraped tissue was then digested with 2 mg/ml collagenase II (c6885; Sigma) for 2 h. The PASMCs were plated in Dulbecco’s modified Eagle’s medium, which contained 20% fetal bovine serum and 1% penicillin and streptomycin. PASMCs were collected as previously described^38^.

### Statistical analysis

All values are denoted as the mean ± SEM. Statistical analysis was performed using Student’s *t*-test or one-way analysis of variance followed by Dunnett’s test where appropriate. A value of *P* < 0.05 was considered statistically significant.

## Electronic supplementary material

Supplementary information
