# Supplementary material for: Granzyme B deficiency promotes osteoblastic differentiation and calcification of vascular smooth muscle cells in hypoxic pulmonary hypertension
Source: Cell Death Dis. 2018 Feb 14;9(2):221. doi: 10.1038/s41419-018-0315-5 (PMC5833422; doi:10.1038/s41419-018-0315-5)
Supplement: Supplementary file 1 — Supplementary information [file 41419_2018_315_MOESM1_ESM.docx]

**Online data supplement**

**Antibodies and reagents**

The antibodies and reagents used were as follows: granzyme B (2C5; Santa Cruz Biotechnology, sc-8022, Texas, U.S.A), Runx2(Abcam, ab23981, MA, U.S.A), MSX2(M-70; Santa Cruz Biotechnology, sc-15396, Texas, U.S.A), BMP2(Abcam, ab14933, MA, U.S.A), SOX9(Abcam, ab26414, MA, U.S.A), SM22α(Abcam, ab14106, MA, U.S.A), Alizarin red S(Sigma, A5533, MO, U.S.A),verapamil HCL(Sigma, 1711202, MO, U.S.A), Ranolazine 2HCL(Sigma, 1598744, MO, U.S.A), HC067047(Sigma, SML0143, MO, U.S.A), IWP-2(Selleck, S7085, TX, U.S.A), STIM1(CDN3H4; Santa Cruz Biotechnology, sc-66173, Texas, U.S.A), ORAI1(H-46; Santa Cruz Biotechnology, sc-68895, Texas, U.S.A), Wnt-5a(A-5; Santa Cruz Biotechnology, sc-365370, Texas, U.S.A), p-p38(D-8; Santa Cruz Biotechnology, sc-7973, Texas, U.S.A), p38(H-147; Santa Cruz Biotechnology, sc-7149, Texas, U.S.A), p-JNK1/2(9H8; Santa Cruz Biotechnology, sc-81502, Texas, U.S.A), JNK1/2(FL; Santa Cruz Biotechnology, sc-571, Texas, U.S.A), p-ERK(E-4; Santa Cruz Biotechnology, sc-7383, Texas, U.S.A), ERK(MK1; Santa Cruz Biotechnology, sc-135900, Texas, U.S.A). Alkaline Phosphatase Diethanolamine Activity Kit (Sigma, AP0100, MO, U.S.A)

**SiRNA and plasmid construction**

The specific siRNA targeted at knocking down HSPA8 was synthesized by Genepharma (Shanghai, China). Non-targeted control siRNA (siNC) was used as negative control. The sequences were as follows, si-HSPA8: 5’- GGCCAGUAUU GAGAUUGAUTT-3’, 3’-TTCCGGUCAUAACUCUAACUA-5’; si-NC: 5’ -UUCUUCGAACGUGUCACGUTT-3’, 3’-TTAAGAGGCGUUCACAGUGCA-5’. The plasmid of GZMB was constructed with GV230 vector and the sequences were as follows: F（519- 539）, CGCAAATGGGCGGTAGGCGTG; R（745- 724）,CGTCGCCGTCCAGCTCGACCAG. The vector alone was taken as negative control. Transfection was implemented according to the manufacturer’s instructions of Lipofectamine® 2000 Reagent (Life technologies, 11668, Carlsbad, CA).

**Morphometric Analysis**

The lung tissues of rats were immobilized in 4% paraformaldehyde for 24 hours, which were then dehydrated and embedded in paraffin wax. For hematoxylin and eosin stain (H&E stain), the paraffin tissue were sliced into 5-um layers and stained, respectively. In immunohistochemistry assay, the 5-um sliced paraffin tissue were dewaxed and restored before incubated with anti-GZMB, anti-HSPA8, and anti-LAMP2A antibodies overnight. The paraffin tissues were washed with PBST and then covered with secondary antibodies for the IgGs. Colored with 3, 3-diaminobenzidine (DAB) and restrained with hematoxylin, tissue sections were recorded by digital photomicrography (Olympus, Japan) as previously described, and analyzed with a color-recognition algorithm of Image-Pro Plus 6.0 as previously described.[^1^](#_ENREF_1)^,^ [^2^](#_ENREF_2)

**Hemodynamic analysis and ventricular weight measurement**

After the animals were anesthetized followed by recording the body weight, a Millar (Millar Instruments Inc, Houston, Tex) catheter was inserted through the right external jugular vein to record RV pressures to evaluate the extent of right ventricular hypertrophy. The heart was removed and the RV free wall was dissected from the left ventricle (LV) plus septum and weighted separately. The degree of right ventricular hypertrophy was determined with the ratio RV/LV+ Septum, as previously described. [^1^](#_ENREF_1)^,^ [^3^](#_ENREF_3)

**Microfil perfusion**

Mice were anesthetized followed by recording the body weight. Fixed with formalin and the lung vasculature were infused with microfil (MV-122, Flow Tech, Inc. [Massachusetts](https://en.wikipedia.org/wiki/Massachusetts), U.S.A). Alcohol-methyl salicylate clearing was implemented to make transparent the lung tissues according to the manufacturer’s instructions.[^1^](#_ENREF_1)

**Echocardiography**

Transthoracic echocardiography was performed on a VisualSonics Vevo 2100 ultrasound machine (FujiFilm VisualSonics Inc) using an MS550D (40 MHz) transducer. Results were calculated using VisualSonics Vevo 2100 analysis software (v. 1.6) with a cardiac measurements package and were based on the average of at least three cardiac cycles. Briefly, mice were anesthetized in an induction chamber filled with 3.5% isoflurane mixed with 100% oxygen and then placed in a supine position on a heated stage containing ECG leads. The heated stage also contained a nosecone filled with 1.5% isoflurane in 100% oxygen delivered at 1.0 L/min and an anal temperature probe by which temperature was maintained at 37°C. A chemical hair remover was used to remove the fur on the chest of the mice. The left ventricle anterior wall thickness during diastole (LVAW) was obtained from the parasternal short axis view using M-mode. The pulmonary artery velocity time integral (PAVTI) were obtained from the parasternal short axis view at the papillary muscle level using M-mode. Pulmonary artery (PA) acceleration time and PA ejection time were obtained from the parasternal short axis view at the aortic valve level using pulsed Doppler mode. All measurements were taken in compliance with the American Society of Echocardiography guidelines.

**Western blot analysis**

The samples were extracted with lysis buffer (Tris 50 mM, pH7.4, NaCl 150 mM, Triton X-100 1%, EDTA 1 mM, and PMSF 2 mM) containing phosphatase inhibitor and incubated for 40 min on ice. The lysates were then sonicated and centrifuged at16,099g for 15 min, and the insoluble fraction was discarded. The protein samples (100–150 μg) were fractionated by SDS-PAGE (12% polyacrylamide gels) and transferred onto nitrocellulose membrane. After incubation in a blocking buffer (Tris 20 mM, pH 7.6, NaCl 150 mM, and Tween 20 0.1%) containing 5% nonfat dry milk powder, Anti-CBFA1(1:400), anti-Msx2(1:400), anti-Bmp2(1:400), anti-STIM1(1:400), anti-ORAI1(1:400), anti-SOX9(1:400), anti-SM22α(1:400) were used as primary antibodies and were incubated overnight at 4℃, followed by reacted with appropriate horseradish peroxidase-conjugated secondary antibodies and enhanced chemiluminescence reagents. β-actin (1:6000) was used as an internal control.

**MTT assay**

PASMCs were cultured at a density of 5000 cells/well in a 96-well culture plate. At the end of treatment in 37°C, the cells were incubated for 4h in a medium containing 0.5% 3-[4,5-dimethylthiazol-2-yl]-2,5-diphenyl-tetrazolium bromide (MTT), the yellow mitochondrial dye. The amount of blue formazan dye formed from MTT is proportional to the number of survival cells. The reaction was terminated with 150 µl DMSO for 10 min. Absorbance at 540 nm was recorded by an enzyme-linked immunosorbent assay plate reader.

**Quantitative RT-PCR**

Total RNA was isolated with TRIzol reagent (Invitrogen, Carlsbad, CA). cDNA was synthesized from 2 μg of RNA with oligo(dT) 18 primers with the use of the Super Script First-Stand cDNA Synthesis Kit(Invitrogen). High-capacity cDNA Reverse Transcription Kit and Fast SYBR® Green Master Mix (Applied Biosystems) were used following the manufacturer’s instructions. RT-PCR was performed on a thermos cycler ABI 7500 fast (Applied Biosystems) for 40 cycles. 18s was used as an internal control. The gene-specific primers were designed from coding regions of that from Gen-Bank TM database. Primer sequences were as follows: Runx2 (rat, [NM_001278483.1](http://www.ncbi.nlm.nih.gov/nuccore/NM_017101.1)), 5’-GAAATAGGCATCAGACAAA-3’ and 5’- AGTAGCAAACCGAAACAC-3’; Runx2 (mice, [NM_0015920](http://www.ncbi.nlm.nih.gov/nuccore/NM_017101.1).2), 5’-AGGACAGCGACTTCATTC-3’ and 5’-CACCTACCAGCCTCACCA-3’; MSX2 (rat, [NM_976515](http://www.ncbi.nlm.nih.gov/nuccore/NM_017101.1).1), 5’-CACGTTCGAGTCCCTAAT-3’ and 5’- GTGTCTTGTAACTGTTGCCTAT-3’; MSX2 (mice, [NM_013601.2](http://www.ncbi.nlm.nih.gov/nuccore/NM_017101.1)), 5’-TGTATCAAGTGGCCCTGT-3’ and 5’-TGGCATGTACCATCTATCC-3’; BMP2 (rat, [NM_250876.1](http://www.ncbi.nlm.nih.gov/nuccore/NM_017101.1)), 5’-GGCTGTGGCAGGCTTTAT-3’ and 5’- TGTCCCTACTGATGAGTTTCT-3’; BMP2 (mice, [NM_084651.1](http://www.ncbi.nlm.nih.gov/nuccore/NM_017101.1)), 5’-GTTCCTCCACGGCTTCTT-3’ and 5’-TGTACCGCAGGCACTCAG-3’; SOX9 (rat, [NM_080403.1](http://www.ncbi.nlm.nih.gov/nuccore/NM_017101.1)), 5’-TGAAGGTGGAGTAGAGCC-3’ and 5’- TCCTACCCAACCATCACG-3’; SOX9 (mice, [NM_011448.4](http://www.ncbi.nlm.nih.gov/nuccore/NM_017101.1)), 5’-ATGCGGGTACTGGTCTGC-3’ and 5’-AAGGGCTACGACTGGACG-3’; SM22α (rat, [NM_031549.2](http://www.ncbi.nlm.nih.gov/nuccore/NM_017101.1)), 5’-CTCTGCACTGCTGCCATA-3’ and 5’-AGTGAAGGTGCCTGAGAAC-3’; 18s (rat, [NM_04623.1](http://www.ncbi.nlm.nih.gov/nuccore/NM_017101.1)), 5’- TTTGACTCAACACGGGAAACC-3’ and 5’- CACGGAATCGAGAAAGAGCTATC-3’; 18s (mice, [NM_003278.3](http://www.ncbi.nlm.nih.gov/nuccore/NM_017101.1)), 5’- AACTTTCGATGGTAGTCGCCG-3’ and 5’- CCTTGGATGTGGTAGCCGTTT-3’.

**Co-immunoprecipitation**

Cells were lysed in RIPA lysis buffer with complete protease inhibitor. Whole-cell lysates were used for co-immunoprecipitation with the indicated antibodies. Generally, 5μg target antibody or IgG was added to 0.5 ml of cell lysate and incubated at 4 °C for 4-6 h. Protein A+G agarose beads were added before the incubation was continued overnight. Immunoprecipitates were washed with lysis buffer and eluted with SDS loading buffer by boiling for 5 minutes. Co-immunoprecipitation data showed the interaction between GZMB and HSPA8.

**Alizarin red S staining**

Alizarin red S staining was used to identify the calcification rate of PASMCs with different treatments. PASMCs were cultured under hypoxia for 7 days upon treatment with procalcifying media containing 2.5mM inorganic phosphate for the induction of calcification. The cells were immobilized with 95% ethanol for 10 min. The cells were stained with alizarin red S dye in 37℃ for 30min.

**Von kossa staining**

Von kossa staining was used to identify the calcification rate of pulmonary arteries with different treatments. Pulmonary arteries were detached from rats. Deposits of calcium mineral was determined followed the manufacturer’s instructions (American Master Tech, KTVKO, MO, U.S.A). Tissue sections were recorded by digital photomicrography (Olympus, Japan)

**Alkaline phosphatase activity**

Alkaline phosphatase activity from pulmonary arteries was assayed followed the manufacturer’s instructions (Sigma, AP0100, MO, U.S.A). Enzyme activity was determined by measuring the cleavage of 10 mM p-nitrophenyl phosphate (pNPP) at 410 nm. Values were normalized to total protein levels, as assessed by the Bio-Rad protein assay reagent (Bio-Rad Laboratories, Hertfordshire, UK).

**Immunofluorescence staining**

PASMCs were cultured on a cover glass (15mm diameter). After treatment, cells were washed three times with PBS, and then fixed with 4% paraformaldehyde at room temperature for 15 min. Permeabilized with 0.03% Triton X-100 for 10min, the cells were blocked with 5% normal bovine serum at 37℃ for 30min. PASMCs were incubated with antibodies (1:50) in PBS at 4 °C overnight. After washed with PBS, the cells were incubated with FITC-conjugated secondary antibody (1:100), Cy3-conjugated IgG secondary secondary antibody (1:100) and DAPI (1:100) away from light. Then the cover glass was mounted and examined with confocal laser scanning microscope (CLSM). The images were merged by CLSM.

**References**

1. Mao M, Yu X, Ge X, Gu R, Li Q, Song S *et al*. Acetylated cyclophilin a is a major mediator in hypoxia-induced autophagy and pulmonary vascular angiogenesis. *J Hypertens* 2017; **35(4)**: 798-809.

2. Ma C, Li Y, Ma J, Liu Y, Li Q, Niu S *et al*. Key role of 15-lipoxygenase/15-hydroxyeicosatetraenoic acid in pulmonary vascular remodeling and vascular angiogenesis associated with hypoxic pulmonary hypertension. *Hypertension* 2011; **58**: 679-688.

3. Shen T, Wang N, Yu X, Shi J, Li Q, Zhang C *et al*. The critical role of dynamin-related protein 1 in hypoxia-induced pulmonary vascular angiogenesis. *J Cell Biochem* 2015; **116**: 1993-2007.

**Supplemental Figure 1**


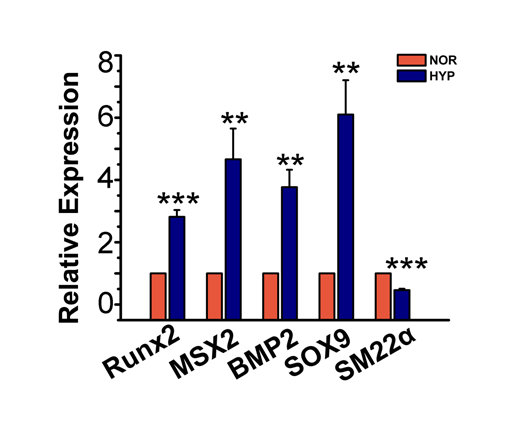


**Supplemental Figure 1.** Real-time PCR of Runx2, MSX2, BMP2, SOX9 expression in pulmonary arteries from normoxia, chronic hypoxia-treated rats. 18s served as the standard; *n*=10.NOR, normoxia; HYP, hypoxia.

**Supplemental Figure 2**


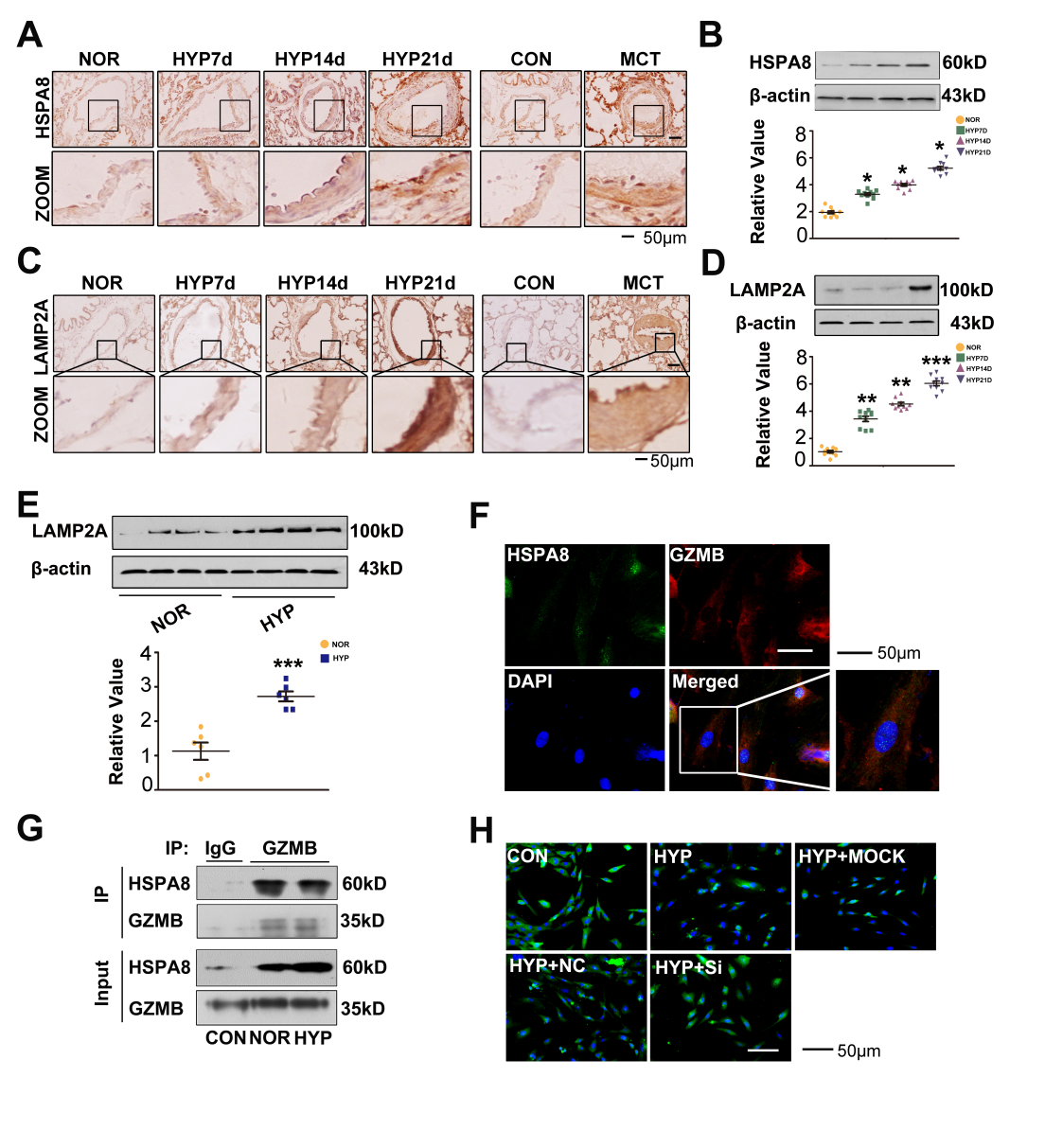


**Supplemental Figure 2.** CMA was activated under hypoxia *in vivo* and *in vitro*. (A) Cellular location of HSPA8 was analyzed immunohistochemically, revealing that it was also located at the medial layer. All panels are at 20× magniﬁcation except for the zoomed ones. Scale bars=50 μm; *n*=10. (B) Expression of HSPA8 was evaluated with western blotting in lung tissues from the normoxia and chronic hypoxia treated rats. β-Actin served as the standard; *n*=10. (C) The cellular location of LAMP2A was analyzed with immunohistochemical, revealing LAMP2A was also located at the medial layer. All panels are at 20× magniﬁcation except for the zoomed ones. Scale bars=50 μm; *n*=10. (D) Expression of LAMP2A was evaluated with western blotting in lung tissues from the normoxia and chronic hypoxia treated rats. β-actin served as the standard; *n*=10. (E) PASMCs were exposed under hypoxia for 24 h and expression of LAMP2A was evaluated with western blotting. β-actin served as the standard; *n*=6. (F) PASMCs were exposed to hypoxia for 24 h and colocalization between GZMB and HSPA8 was determined with immunofluorescence. HSPA8 (green), GZMB (red) and DAPI (blue). All panels are at 40× magniﬁcation; *n*=6. (G) PASMCs were exposed to hypoxia for 24 h and the whole cell lysates were extracted for co-immunoprecipitation with anti-GZMB, followed by probing with anti-HSPA8; *n*=6. (H) Expression of HSPA8 was knocked down with specific targeted siRNA under hypoxia and activity of GZMB was estimated with immunofluorescence staining; *n*=6. Data are represented as mean±SEM. *P*<0.05*，0.01** and 0.001*** versus normoxia group. *P*<0.05#, 0.01## and 0.001### versus hypoxia+negative control group. NOR, normoxia; HYP, hypoxia; MCT, monocrotaline.

**Supplemental Figure 3**

**
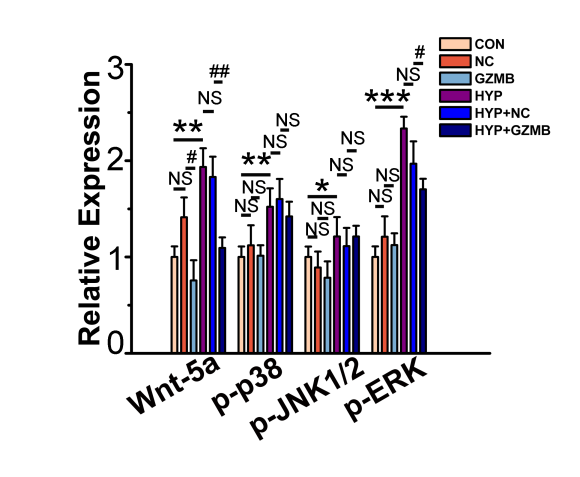
**

**Supplemental Figure 3.** Overexpression of GZMB was achieved with plasmid and several classical signaling pathways were evaluated with western blotting. β-Actin served as the standard for Wnt-5a; Total p38, JNK1/2, ERK served as the standard for p-p38, p-JNK1/2 and p-ERK, respectively; *n*=6.

**Supplemental Figure 4**


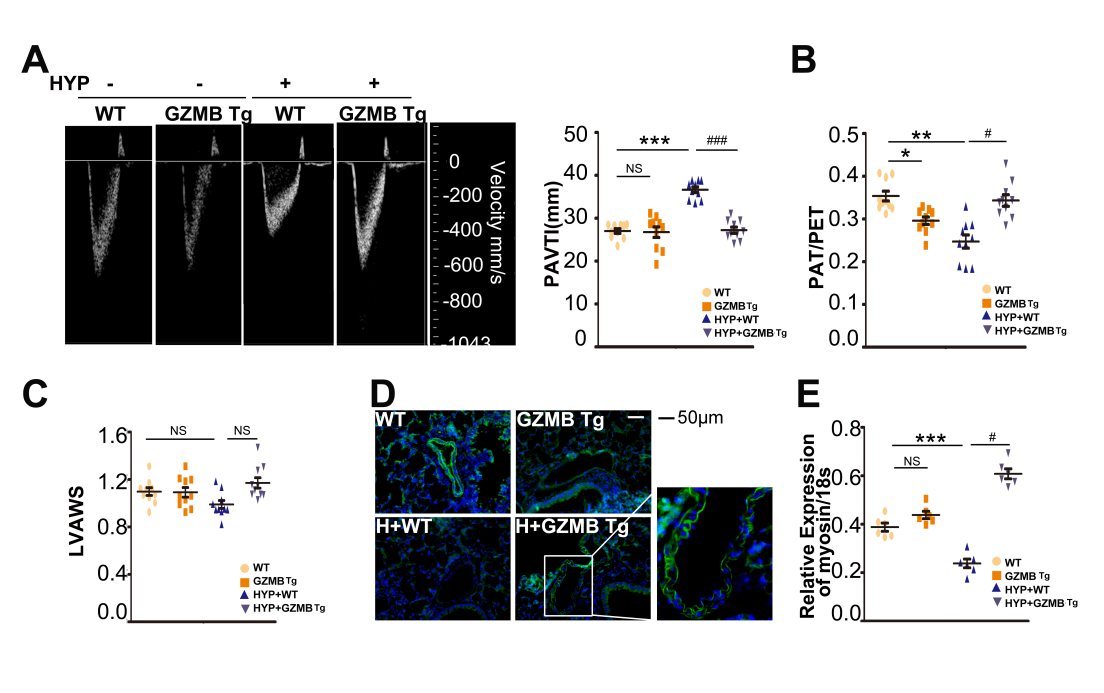


**Supplemental Figure 4.** (A–C) Echocardiography showed decreased RV function in SM22α-GZMB Tg mice (age 8-12 weeks). Increased pulmonary artery velocity time integral (PAVTI; A) and decreased pulmonary artery acceleration time/pulmonary artery ejection time (PAT/PET, B) ratio were blocked in SM22α-GZMB Tg mice under hypoxia. (C) Change in LV anterior wall thickness (LVAWS) between wild-type and SM22α-GZMB Tg mice was not significant. *n*=10; (D) Expression of myosin was estimated with immunofluorescence staining in lung tissues from wild-type and SM22α-GZMB Tg mice after hypoxia and normoxia for 3 weeks; *n*=6. (E) Real-time PCR estimated mRNA level of myosin; 18 s served as the standard; *n*=6. Data are represented as mean±SEM. *P*<0.05*, 0.01** and 0.001*** versus wild type group. *P*<0.05^#^, 0.01^##^ and 0.001^###^ versus hypoxia+wild-type group. WT, wild type; HYP, hypoxia.

**Supplemental Figure 5**


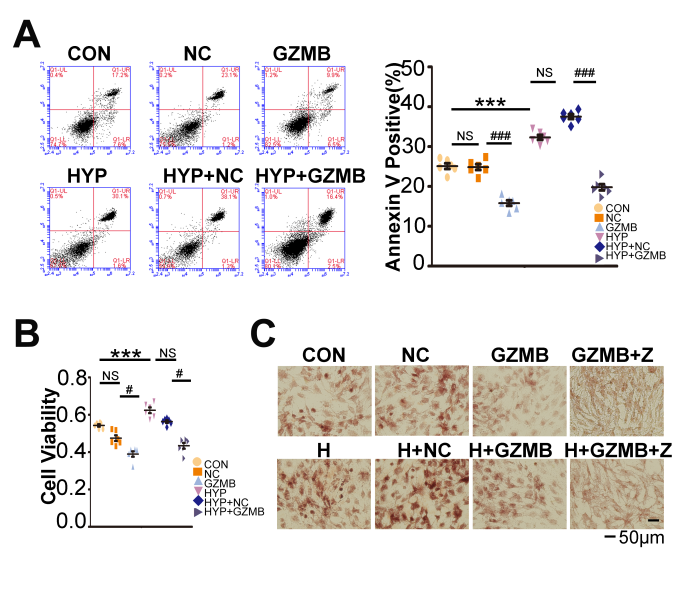


**Supplemental Figure 5**. GZMB was capable of inducing apoptosis but this effect is not related to its role in PASMC’s mineralization. (A) Apoptosis analysis by flow cytometer showed that the reduction of apoptotic cells caused by hypoxia was reversed by GZMB, *n*=6. (B) Viability of PASMCs was evaluated by MTT, GZMB promoted the apoptosis; *n*=6. (C) Z-VAD was administrated to inhibit the apoptosis and PASMCs were cultured under hypoxia for 7 days upon treatment with procalcifying media containing 2.5mM inorganic phosphate. Deposits of calcium mineral were assessed by Alizarin Red S staining. Inhibiting apoptosis did not reverse the reduced PASMC’s mineralization by GZMB; *n*=5. Data are represented as mean±SEM. *P*<0.05*,0.01** and 0.001***versus normoxia group. *P*<0.05#, 0.01## and 0.001### versus hypoxia+negative control group. CON, control; HYP, hypoxia; GZMB, granzyme B. NC, negative control; Z, Z-VAD.

**Supplemental Figure 6**

**
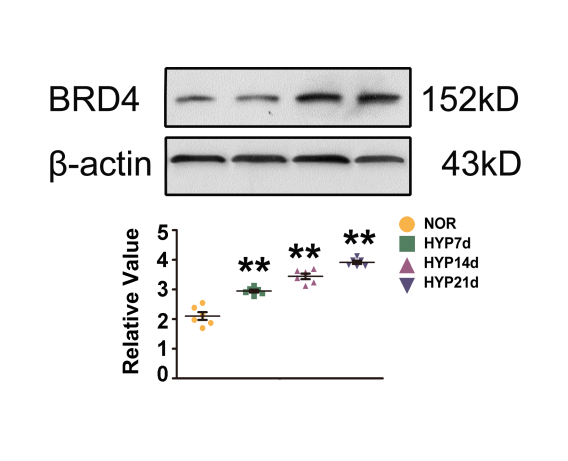
**

**Supplemental Figure 6**. BRD4 was activated under hypoxia *in vivo*. Expression of BRD4 was evaluated with western blotting in lung tissues from the normoxia and chronic hypoxia treated rats. β-Actin served as the standard; *n*=6. Data are represented as mean±SEM. *P*<0.01**versus normoxia group. NOR, normoxia; HYP, hypoxia.

**Supplemental Figure 7**

**
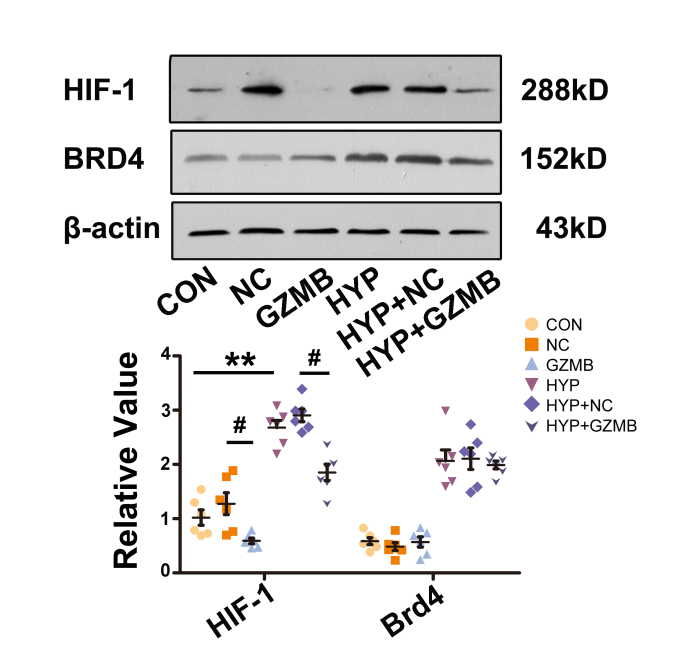
**

**Supplemental Figure 7**. Plasmid aimed at overexpression of GZMB was transfected into PASMCs and then exposed to hypoxia for 24 h. Expression of HIF-1 and BRD4 were evaluated with western blotting. β-Actin served as the standard; *n*=6. Data are represented as mean±SEM. *P*<0.01** versus normoxia group. *P*<0.05^#^versus negative control group. CON, control; HYP, hypoxia; GZMB, granzyme B plasmid; NC, negative control.

**Supplemental Figure 8**


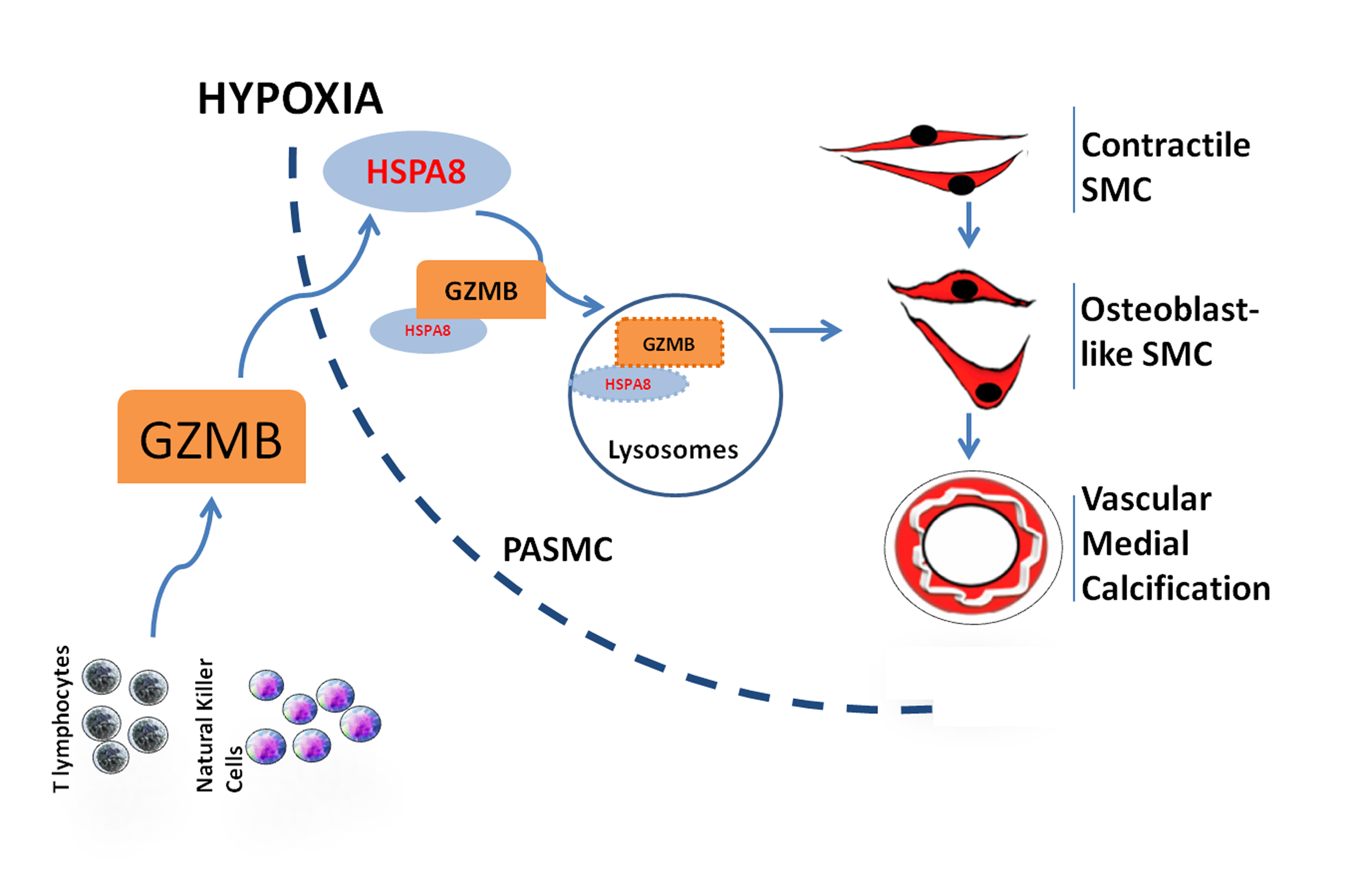


**Supplemental Figure 8. A schematic model showing the proposed mechanism for the role of GZMB in vascular calcification of PAH.** GZMB was specifically recognized and degraded by activation of CMA process, which promoted the osteoblastic differentiation and calcification of PASMCs, resulting in the vascular calcification of PAH. “GZMB” granzyme B.
